# Neural Circuitry Underlying *Drosophila* Female Postmating Behavioral Responses

**DOI:** 10.1016/j.cub.2012.04.062

**Published:** 2012-07-10

**Authors:** Carolina Rezával, Hania J. Pavlou, Anthony J. Dornan, Yick-Bun Chan, Edward A. Kravitz, Stephen F. Goodwin

**Affiliations:** 1 Department of Physiology, Anatomy and Genetics, University of Oxford, Sherrington Building, Parks Road, Oxford OX1 3PT, UK; 2 Institute of Molecular Cell & Systems Biology, College of Medical, Veterinary and Life Sciences, University of Glasgow, Glasgow G12 8QQ, UK; 3 Department of Neurobiology, Harvard Medical School, Boston, MA 2115, USA

## Abstract

**Background:**

After mating, *Drosophila* females undergo a remarkable phenotypic switch resulting in decreased sexual receptivity and increased egg laying. Transfer of male sex peptide (SP) during copulation mediates these postmating responses via sensory neurons that coexpress the sex-determination gene *fruitless* (*fru*) and the proprioceptive neuronal marker *pickpocket* (*ppk*) in the female reproductive system. Little is known about the neuronal pathways involved in relaying SP-sensory information to central circuits and how these inputs are processed to direct female-specific changes that occur in response to mating.

**Results:**

We demonstrate an essential role played by neurons expressing the sex-determination gene *doublesex* (*dsx*) in regulating the female postmating response. We uncovered shared circuitry between *dsx* and a subset of the previously described SP-responsive *fru^+^*/*ppk^+^-*expressing neurons in the reproductive system. In addition, we identified sexually dimorphic *dsx* circuitry within the abdominal ganglion (Abg) critical for mediating postmating responses. Some of these *dsx* neurons target posterior regions of the brain while others project onto the uterus.

**Conclusions:**

We propose that *dsx*-specified circuitry is required to induce female postmating behavioral responses, from sensing SP to conveying this signal to higher-order circuits for processing and through to the generation of postmating behavioral and physiological outputs.

## Highlights

► *dsx* circuitry plays a pivotal role in the female postmating switch ► Peripheral *dsx* neurons detect and respond to sex peptide ► Central *dsx* neurons convey this signal to higher-order processing and direct postmating responses.

## Introduction

In *Drosophila*, genes of the sex-determination hierarchy orchestrate the development and differentiation of sex-specific tissues, establishing sex-specific physiology and neural circuitry. *doublesex* (*dsx*) and *fruitless* (*fru*), two pivotal transcription factors of the sex determination hierarchy, establish most aspects of “maleness” and “femaleness” [1]. While *fru/dsx-*expressing neurons have been shown to regulate male courtship behaviors [2–5], less is known about the circuitry underlying female mating. Prior to copulation, *Drosophila* females must assess potential mates for species type and fitness before sanctioning mating [1]. An unreceptive female exhibits rejection behaviors such as kicking and ovipositor extrusion [1, 6–8]. A receptive female will facilitate copulation by slowing down, ceasing rejection behaviors, and opening her vaginal plates [1]. While virgin females are highly receptive and rapidly copulate with a suitable partner, mated females undergo a substantial change in their physiology and behavior, becoming temporarily sexually unreceptive to further copulatory attempts, while increasing oviposition [9].

These postmating responses are triggered primarily by sex peptide (SP), a small peptide synthesized in the male accessory glands and transferred to the female during insemination [9]. Females mated to SP-deficient males readily mate again [10, 11], while virgin females injected with SP are rendered unreceptive [12]. Recently, a SP-responsive G protein-coupled receptor for SP-mediated postmating responses has been identified [13]. Females lacking this sex peptide receptor (SPR) remain receptive, exhibiting virgin-like behaviors, postmating. It was shown that postmating responses could be mediated by SPR activation in a set of six to eight sensory neurons located on the reproductive tract [14, 15]. These neurons express both the sex-specific transcripts of the *fruitless* gene (*fru*^+^), a marker for neurons likely to have sex-specific functions, and *pickpocket* (*ppk^+^*), which is believed to encode a marker for proprioceptive neurons [14–17].

How higher-order neural circuits respond to stimuli from these receptor inputs to coordinate the change from pre- to postcopulatory states in females is unknown. As these *fru^+^*/*ppk^+^* sensory neurons project to the Abg and may target the subesophageal ganglion (SOG), these regions in the central nervous system (CNS) are proposed to be involved in processing of SP-sensory information [14, 15]. The interneurons involved in transmission of this information and the specific neural loci responsible for regulation of these postmating behaviors together with their associated motor outputs remain to be determined.

Disrupting synaptic activity of *dsx^Gal4^*-expressing neurons in females impairs specific courtship behaviors [4]; these virgin females are incapable of sampling the male's courtship displays and therefore incapable of providing acceptance responses. During copulation, this lack of cooperation is evident by continual female movement and rejection behaviors. Although sperm and seminal fluids are transferred, these females lay no eggs, remate, and remain incapable of suppressing further courtship, indicating that *dsx* neurons are involved in both pre- and postcopulatory female reproductive behaviors [4]. The suppression of postmating responses via disruption of *dsx* neuronal function [4] also suggested an overlap between *dsx* and the SP-responsive *fru^+^*/*ppk^+^*-expressing neurons in the reproductive system.

In this study we confirm the direct involvement of *dsx* neural circuitry in female postmating responses, demonstrating that SP expression in *dsx* neurons is sufficient to induce a postmating behavioral switch in virgin females. In addition, SPR expression in *dsx* neurons alone is sufficient for SP-induced postmating behaviors. We observed *dsx* expression in most sensory neurons of the reproductive system, including a subpopulation of the previously characterized *fru^+^*/*ppk^+^* neurons located in the uterus, further refining the sensory circuitry underlying the postmating switch. These results indicate that *dsx* and *fru* shared circuitry is required for eliciting female postmating responses.

Using an intersectional Flippase-recombinase approach to manipulate subsets of *dsx* neurons, we uncovered downstream *dsx* neuronal elements associated with this *dsx*^+^/*fru*^+^/*ppk*^+^ uterine sensory cluster in the CNS involved in mediating postmating responses. These *dsx* neurons in the Abg project to specific locations within the brain and the reproductive system. Our findings indicate that *dsx* circuitry consists of not only primary neurons required for sensing SP in the reproductive system, but also downstream effectors of the SP-sensory neuronal pathway that regulate postcopulatory behavior of females. We propose that *dsx* neurons in the Abg act as relay neurons, transmitting SP information from the reproductive system to decision centers in the brain, and that additional *dsx* neurons in this area direct postmating behaviors such as egg transport and oviposition.

## Results

### Targeted Expression of Membrane-Bound SP in *doublesex*-Expressing Neurons Triggers Postmating Behaviors

**Figure 1:**
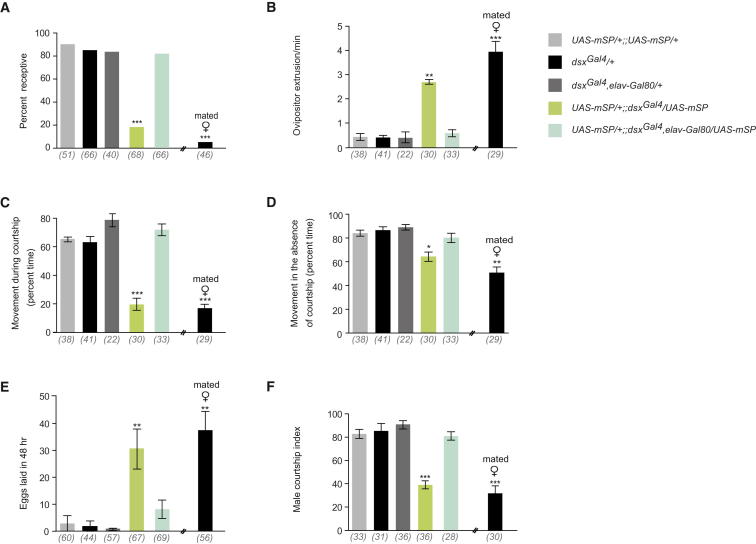
Behavioral Effects of mSP Expression in *dsx* Neurons in Virgin Females (A) Percentage receptivity (^∗∗∗^p < 0.0001, Fisher exact test). (B) Ovipositor extrusion during courtship (^∗∗^p < 0.001, ^∗∗∗^p < 0.0001, Kruskal-Wallis ANOVA test). (C) Percentage of time spent moving while being actively courted (^∗∗∗^p < 0.0001, Kruskal-Wallis ANOVA test). (D) Percentage of time spent moving in the absence of active courtship (^∗^p < 0.05, ^∗∗^p < 0.001, Kruskal-Wallis ANOVA test). (E) Egg laying (^∗∗^p < 0.001, Kruskal-Wallis ANOVA test). (F) Male courtship index of naive males (^∗∗∗^p < 0.0001, Kruskal-Wallis ANOVA test). Error bars indicate SEM. Genotypes indicate virgin females, unless stated otherwise. Target males were wild-type. n values shown in parentheses.

Expression of a membrane-bound form of SP (mSP) in the CNS has been shown to be sufficient to induce postmating responses in virgin females and has been used to identify SP-responsive cells and tissues [15, 18]. Employing a similar strategy, we targeted expression of *UAS*-*mSP* to *dsx* cells under the control of *dsx^Gal4^* in virgin females, allowing us to assess the role of such cells in sensing SP. These virgin females exhibited levels of receptivity comparable to mated control females (<20% copulated within 1 hr), involving levels significantly lower than those of control virgin females (>80% copulated within 1 hr) (Figure 1A). As a corollary, *dsx^Gal4^*/*UAS*-*mSP* virgin females displayed significant levels of rejection behaviors toward courting males, as demonstrated by the increased frequency of ovipositor extrusions (Figure 1B and [6]). We also observed an increase in oviposition in *dsx^Gal4^*/*UAS*-*mSP* virgin females, similar to levels observed with mated controls (Figure 1E).

Studies have shown the importance of female locomotor activity for stimulating male courtship [19–21]. Consistent with this, we observed increased locomotor activity in virgin females while being courted compared with mated wild-type females (Figure 1C). Tellingly, *dsx^Gal4^*/*UAS*-*mSP* virgin females displayed a marked decrease in locomotion while being courted (Figure 1C). It has been suggested that a female's movement could produce subtle sounds alerting males to the female's presence and initiating courtship behaviors [21]. During courtship observation periods, both *dsx^Gal4^*/*UAS*-*mSP* virgin females and mated control females exhibited reduced levels of activity while the male was not courting, remaining static for significantly greater periods of time than control virgin females (Figure 1D). These results show that locomotion during courtship is another behavior that undergoes a dynamic change in response to the copulatory “state” of an individual female.

The male courtship index (CI) is a measure of the attractiveness of a female: naive males court virgin females persistently but display lower levels of courtship when paired with unreceptive mated females [1]. Intriguingly, wild-type naive males courted *dsx^Gal4^/UAS*-*mSP* virgin females significantly less than control virgin females (<40% versus > 80%, Figure 1F). To verify that the observed decrement in male CIs was a consequence of female behavior rather than a response to potential changes in the experimental female's pheromonal profile, we repeated these experiments with decapitated (i.e., nonbehaving) *dsx^Gal4^*/*UAS-mSP* virgin females. We observed that the males' CIs were indistinguishable to those displayed in the presence of wild-type virgins (Figure S1A available online). This suggests that the male's courtship behavioral outputs are undergoing dynamic modification in response to the target female's behavioral state.

We confirmed the neural etiology of the observed defects in postmating responses in *dsx^Gal4^*/*UAS*-*mSP* virgin females by employing *elav*-*Gal80* [4] specifically to inhibit neuronal expression of mSP. *elav*-*Gal80; dsx^Gal4^*/*UAS*-*mSP* virgin females were not significantly different from controls (Figures 1A–1F).

During our analyses of *dsx^Gal4^*-driven nGFP expression in conjunction with *elav*-*Gal80* we noted repression in nonneuronal cells in the reproductive system (Figure S2B). To ensure that this repression did not impinge upon the neural etiology of the observed *dsx^Gal4^*/*UAS*-*mSP* phenotypes, we reiterated these experiments using an alternative panneuronal repressor, *syb-Gal80* [22] (Figure S2C). *dsx^Gal4^*-driven mSP expression in conjunction with *syb*-*Gal80* yielded behavioral results consistent with those observed using *elav*-*Gal80*; that is, receptivity levels were restored in *dsx^Gal4^*/*UAS*-*mSP* coexpressing *syb-Gal80* to ∼90% (n = 34), and egg laying was reduced to control levels (7.5 ± 2.4, n = 45).

These results demonstrate that expression of mSP in *dsx* neurons is sufficient to induce female postmating behaviors in virgin females, and these changes can engender complex behavioral responses in individual females as well as in potential mates.

### Knocking Down SPR Function Specifically in *dsx*-Expressing Neurons Abolishes Postmating Behavioral Responses

**Figure 2:**
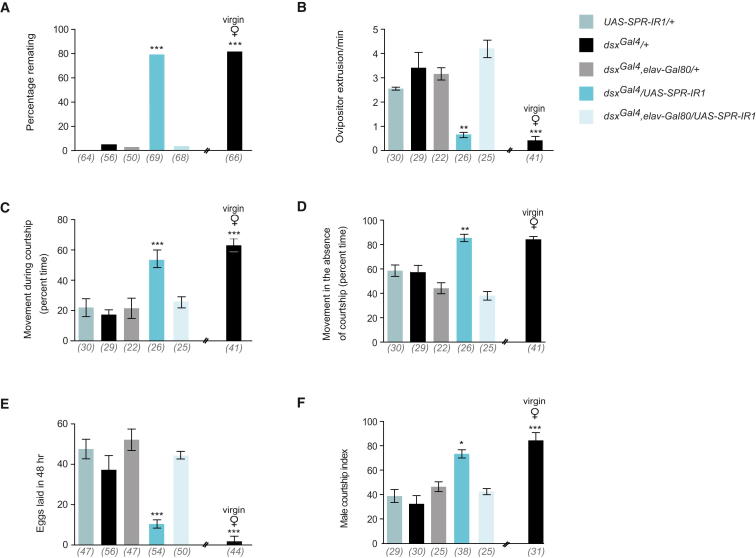
Knocking Down SPR Expression in *dsx* Neurons Reduces Postmating Behaviors in Mated Females (A) Percentage remating (^∗∗∗^p < 0.0001, Fisher exact test). (B) Ovipositor extrusion during courtship (^∗∗^p < 0.001, ^∗∗∗^p < 0.0001, Kruskal-Wallis ANOVA test). (C) Percentage of time spent moving while being actively courted (^∗∗∗^p < 0.0001, Kruskal-Wallis ANOVA test). (D) Percentage of time spent moving in the absence of active courtship (^∗∗^p < 0.001, Kruskal-Wallis ANOVA test). (E) Egg laying (^∗∗∗^p < 0.0001, Kruskal-Wallis ANOVA test). (F) Male courtship index of naive males (^∗^p < 0.05, ^∗∗∗^p < 0.0001, Kruskal-Wallis ANOVA test). Error bars indicate SEM. Genotypes indicate mated females, unless stated otherwise. Target males were wild-type. n values shown in parentheses.

SPR is broadly expressed in the CNS and reproductive tract ([13] and Figure S3) and is required for SP-mediated postmating behavioral responses, as females lacking SPR remain receptive, exhibiting virgin-like behaviors, even after mating or SP injection [13]. To show that *dsx^Gal4^*-expressing cells respond to SP, we employed RNA interference to knock down SPR expression [13, 14] and evaluated the effects on postmating responses. While virgin *dsx^Gal4^*/*UAS-SPR-IR1* females were typically receptive (data not shown), after mating they displayed no decrement in receptivity nor any increase in ovipositor extrusions compared with mated controls (∼80% versus < 4% remated, respectively; Figures 2A and 2B). *dsx^Gal4^*/*UAS-SPR-IR1* mated females displayed locomotor activity comparable to virgin females (Figures 2C and 2D) and significantly decreased levels of oviposition (Figure 2E). Males spent significantly more time courting *dsx^Gal4^*/*UAS-SPR-IR1* mated females compared to controls (CI > 60% versus < 40%, respectively; Figure 2F). These effects were not observed when males were paired with decapitated *dsx^Gal4^*/*UAS-SPR-IR1* females (Figure S1B).

**Figure 3:**
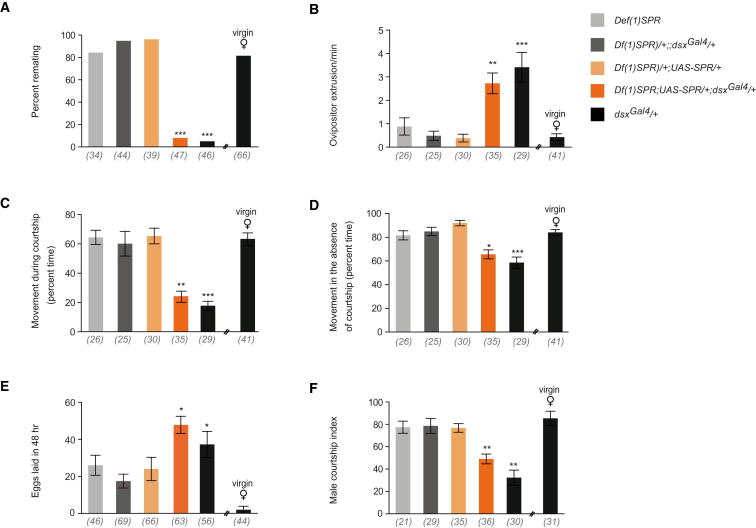
Expression of SPR in *dsx* Cells Alone Is Sufficient to Rescue Postmating Responses in *SPR*-Deficient Line *Df(1)SPR* (A) Percentage remating (^∗∗∗^p < 0.0001, Fisher exact test). (B) Ovipositor extrusion during courtship (^∗∗^p < 0.001, ^∗∗∗^p < 0.0001, Kruskal-Wallis ANOVA test). (C) Percentage of time spent moving while being actively courted (^∗∗^p < 0.001, ^∗∗∗^p < 0.0001, Kruskal-Wallis ANOVA test). (D) Percentage of time spent moving in the absence of active courtship (^∗^p < 0.05, ^∗∗∗^p < 0.0001, Kruskal-Wallis ANOVA test). (E) Egg laying (^∗^p < 0.05, Kruskal-Wallis ANOVA test). (F) Male courtship index of naive males (^∗∗^p < 0.001, Kruskal-Wallis ANOVA test). Error bars indicate SEM. Genotypes indicate mated females, unless otherwise stated. Target males were wild-type. n values shown in parentheses.

We then attempted to rescue the behavioral deficits observed in SPR null mated females by restrictively expressing SPR in *dsx*-expressing cells in a SPR-deficient genetic background [*Df(1)SPR*]. Postmating responses were restored in *Df(1)SPR*;*UAS-SPR;dsx^Gal4^* mated females. Rescued females exhibited significantly reduced levels of receptivity, increased ovipositor extrusion, egg laying, and decreased locomotion; they also elicited lowered levels of male courtship (Figures 3A–3F). Decapitated *Df(1)SPR*;*UAS-SPR;dsx^Gal4^* mated females were found to be as attractive as decapitated mated control females to naive males (Figure S1C). We used *elav*-*Gal80* to confirm the neural etiology of phenotypic effects arising from impairment of SPR function in *dsx^Gal4^*-expressing neurons. Expression of *elav*-*Gal80* in conjunction with *dsx^Gal4^*/*UAS-SPR-IR1* in mated females rescued all observed behavioral deficits, including percentage of females remating and levels of egg laying (Figures 2A–2F).

These findings demonstrate the necessity and sufficiency of SPR function in *dsx* neurons to respond to, and convey, the stimulatory cues engendered by SP, resulting in the induction of postmating behavioral responses.

### *dsx* Neurons Are Part of the *fru*^+^/*ppk*^+^ Circuitry Involved in Sensing SP

Although SPR is detected broadly on the female reproductive tract and superficial regions of the ventral nerve cord (VNC) and brain [13], only a restricted subset of *fru*^+^/*ppk*^+^ sensory neurons expressing SPR in the reproductive system appear necessary and sufficient for inducing SP-mediated postmating responses [14, 15].

**Figure 4:**
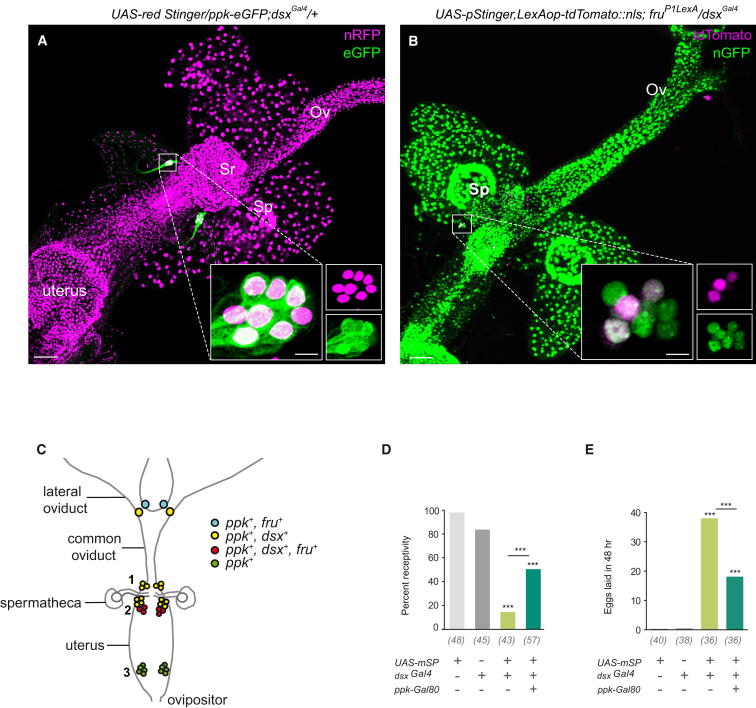
*dsx* Neurons Are Part of the Circuitry Involved in Sensing SP (A) Colocalization of *ppk*- and *dsx-*expressing neurons in the female genital tract. *ppk-eGFP* was used to visualize *ppk* neurons (green), while *dsx-*expressing cells were visualized with *dsx^Gal4^* and *UAS*-*redStinge*r (nRFP; magenta). Inset in (A) shows overlap between *ppk*- and *dsx-*expressing neurons at higher optical magnification. (B) Colocalization of *fru*- and *dsx-*expressing neurons in the female genital tract. *fru* neurons were visualized with *LexAop-tdTomato::nls* under the control of *fru^P1LexA^* (tdTomato; magenta); *dsx* neurons were visualized with *dsx^Gal4^* and *UAS*-*pStinge*r (nGFP; green). Inset in (B) shows overlap between *fru*- and *dsx-*expressing neurons at higher magnification. (A and B) Scale bars represent 50 μm and 10 μm in insets. Seminal receptacle (Sr), spermathecae (Sp), and common oviduct (Ov) are indicated. (C) Schematic representation of the *Drosophila* female reproductive system showing different clusters of sensory neurons expressing *ppk*, *fru* and/or *dsx*. Each lateral oviduct possesses two *ppk-*expressing neurons [15], one of which is *fru*^+^ (blue dots), while the other is *dsx*^+^ (yellow dots). Within the uterus, the most anterior cluster (no. 1) comprises three bilateral *dsx^+^/ppk^+^* neurons (yellow dots); the more posterior cluster (no. 2) comprises seven bilateral *dsx^+^/ppk^+^* neurons, three of which also express *fru* (yellow and red dots respectively); while the most posterior cluster in the uterus (no. 3) appears to solely express *ppk* (green dots). (D and E) *ppk*-*Gal80* expression reduces postmating behavioral responses in *dsx^Gal4^*/*UAS*-*mSP* virgin females. (D) Percentage receptivity (^∗∗∗^p < 0.0001, Fisher exact test). (E) Egg laying (^∗∗∗^p < 0.0001, Kruskal-Wallis ANOVA test). Error bars indicate SEM. Genotypes indicate virgin females. Target males were wild-type. n values shown in parentheses.

Our behavioral observations suggest that *dsx* neurons respond directly to SP to elicit the postmating switch. To ascertain whether the *fru*^+^/*ppk*^+^ neurons involved in modulation of postmating responses to SP signaling were also *dsx* positive, we expressed *UAS-redStinger* using *dsx^Gal4^* alongside *ppk-eGFP*. The full population of *ppk* neurons associated with the female genitalia consists of two neurons on each lateral oviduct plus approximately 30 neurons organized in three bilateral clusters on the uterus (see Figure 4C; [15]). Colocalization was observed with *dsx* in most *ppk* sensory neurons; this included a pair of neurons on the lateral oviducts and the two most anterior bilateral clusters on the uterus (Figures 4A, 4C1, and 4C2). In agreement with previous characterizations, the most anterior clusters (Figure 4C1) on the uterus are bilateral clusters comprising three neurons, while the more posterior clusters (Figure 4C2) are bilateral clusters comprising seven neurons [14, 15]. Further support that *dsx*^+^/*ppk*^+^ neurons are required for activating the postcopulatory switch was evidenced by the reduction in postmating responses observed when *dsx^Gal4^* drove expression of mSP in virgin females in the presence of *ppk-Gal80* [15]; Figures 4D and 4E).

A specific subset of *ppk* neurons, one per lateral oviduct and a bilateral cluster of three neurons in the uterus, has been shown to coexpress with marker expression driven by *fru^Gal4^* [14, 15]. We exploited the LexA/lexAop binary system [23] to confirm coexpression between *dsx* and *fru* using *UAS-pStingerII* (nGFP) and *lexAop-dsTomato* responsive elements in conjunction with the *dsx^Gal4^* and *fru^P1LexA^* drivers [24]. Coexpression was restricted to three *fru*^+^ neurons on each side of the uterus (Figures 4B and 4C2), with no coexpression observed on the lateral oviducts (data not shown). We have therefore refined the originally described *fru*^+^/*ppk*^+^ SP-sensory circuit to two pivotal bilateral clusters of three *dsx*^+^/*fru*^+^/*ppk*^+^ neurons.

### *dsx* Sensory Neurons in the Female Reproductive System Appear Cholinergic

To investigate the neurochemical properties of the neurons implicated in triggering SP-mediated postcopulatory changes, we behaviorally screened neurotransmitter Gal4 lines driving mSP. Of the lines tested, only *Cha-Gal4* [25] resulted in a reduction in receptivity in virgin females, fewer than 7% of which achieved copulation in 1 hr (Table S1 and Figure S4C). Expression of *Cha-Gal4* driving nGFP in the reproductive system costained with the panneuronal marker anti-ELAV identified neurons that appear anatomically and topographically congruent with the identified *dsx* neurons—that is, two of the three neurons of the anterior bilateral cluster on the uterus (Figure 4C1), all seven neurons of the posterior bilateral cluster on the uterus (Figure 4C2), and one neuron per lateral oviduct (Figures S2A and S4A).

Consistent with these findings, *Cha-Gal80* abolished nGFP expression in all *dsx* neurons within the reproductive system (Figure S4B). Moreover, *dsx^Gal4^*/*UAS*-*mSP* virgin females expressing *Cha-Gal80* demonstrated high levels of receptivity (∼80%) and reduced levels of egg laying (Figures S4C and S4D, respectively). These results suggest that SP-responsive *dsx* neurons may be cholinergic.

### Intersecting *dsx* Neurons Critical for Mediating Postmating Behavioral Responses

Although progress has been made identifying sex-peptide-responsive sensory neurons [14, 15], little is known about the downstream neural circuitry underlying postmating responses. Silencing all *dsx^Gal4^* neurons impairs postmating behaviors, such that mated females failed to lay eggs, remained attractive to courting males, and remated with a second male [4]. *dsx* is expressed in ∼50 neurons in the female brain and ∼300 neurons in the Abg [4]. We found a subpopulation of *dsx-*Abg neurons sending two bilateral fascicles along the abdominal nerve trunks that ramify on the uterus and vaginal plates (Figure 6A), suggesting that *dsx* neurons might play a role downstream of the SP-sensory neurons in mediating postmating responses.

**Figure 5:**
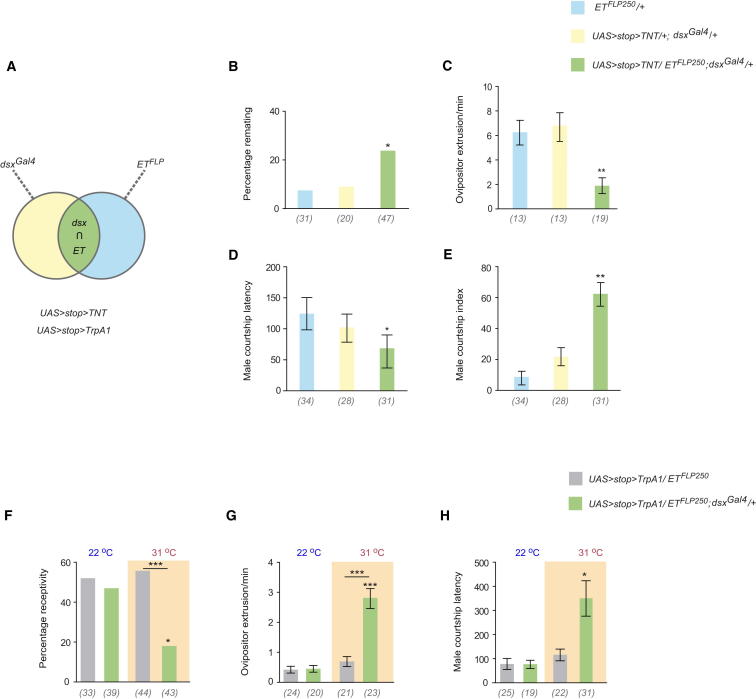
Intersecting *dsx* Neurons Critical for Mediating Postmating Behavioral Responses (A) Schematic of FLP/FRT intersectional strategy employed to subdivide *dsx* circuitry. Crossing an *ET^FLP^* line with *dsx^Gal4^* allows expression of the selected effectors, *UAS* > *stop > TNT or UAS* > *stop > TrpA1*, in all intersecting neurons (ET∩*dsx*) where FLP has excised the transcriptional stop cassette (*>stop >*). (B–E) Effects of silencing intersecting *ET^FLP250^*/*dsx^Gal4^* neurons on postmating behaviors using *UAS* > *stop > TNT*. (B) Percentage remating (^∗^p < 0.01, Fisher exact test). (C) Ovipositor extrusion during courtship (^∗∗^p < 0.001, Kruskal-Wallis ANOVA test). (D) Male courtship latency in seconds (mean ± SEM, ^∗^p < 0.01, Kruskal-Wallis ANOVA test). (E) Male courtship index of naive males (^∗∗^p < 0.001, Kruskal-Wallis ANOVA test). Genotypes indicate mated females. Target males were wild-type. n values shown in parentheses. (F–H) Effects of artificially activating intersecting *ET^FLP250^*/*dsx^Gal4^* neurons on postmating behaviors using *UAS* > *stop > TrpA.* (F) Percentage receptivity (^∗^p < 0.01, ^∗∗∗^p < 0.0001, Fisher exact test). (G) Ovipositor extrusion during courtship (^∗∗∗^p < 0.0001, Kruskal-Wallis ANOVA test). (H) Male courtship latency (^∗^p < 0.01, Kruskal-Wallis ANOVA test). Experiments were performed at the permissive and restrictive temperatures of 22°C and 31°C, respectively. Error bars indicate SEM. Genotypes indicate virgin females. Target males were wild-type. n values shown in parentheses.

We implemented a FLP/FRT intersectional strategy [26] to subdivide the *dsx* circuitry into functionally defined subsets of neurons underlying postmating behaviors. This involves crossing an enhancer-trap FLP (*ET^FLP^*) line with *dsx^Gal4^* and a given reporter (e.g., *UAS > stop > mCD8::GFP*) or *effector (e.g., UAS > stop > TNT*) to express membrane-bound GFP or the synaptic vesicle blocker TNT in neurons in which FLP has excised the transcriptional stop cassette (*> stop >*) (Figure 5A). Screening a collection of *ET^FLP^* lines specific to the nervous system by expressing *UAS > stop > TNT* in combination with *dsx^Gal4^*, we identified one line (*ET^FLP250^*) that showed robust impairment of postmating behavioral responses.

Silencing *ET^FLP250^*/*dsx^Gal4^* intersecting neurons was sufficient to partially recapitulate the defects observed with fully silenced *dsx* circuitry [4]; *ET^FLP250^/UAS > stop > TNT/dsx^Gal4^* mated females did not extrude their ovipositor to prevent copulation (Figure 5C), resulting in a significantly higher percentage of remating compared with mated controls (Figure 5B), and laid significantly fewer eggs in 48 hr than mated controls (29.5 ± 2.2, n = 30 versus 42.2 ± 2.7, n = 30, respectively). These mated females remained highly attractive to males, demonstrated by decreased courtship latency and higher-than-usual levels of elicited courtship (Figures 5D and 5E).

If inhibiting *dsx* neuronal function interrupts postmating behaviors in mated females, then activating these neurons may induce postmating responses in virgin females. To address this, we expressed the heat-activated ion channel TrpA1 (*UAS-TrpA1*; [27]) in *ET^FLP250^*/*dsx^Gal4^* virgin females and assessed the behavioral effects of transient neuronal activation. At the permissive temperature, 22°C, *ET^FLP250^/UAS > stop > TrpA1/ dsx^Gal4^* virgin females behaved indistinguishably from virgin controls (Figures 5F–5H). At the restrictive temperature, 31°C, thermal activation of these neurons resulted in reduced levels of receptivity (Figure 5F) and significantly higher levels of female rejection behaviors toward courting males (Figure 5G). Although levels of male courtship toward these females were unchanged (data not shown), a significant increase in courtship latency was observed (Figure 5H).

### *dsx*-Abg Neurons Involved in Postmating Responses Target the Brain and Reproductive System

**Figure 6:**
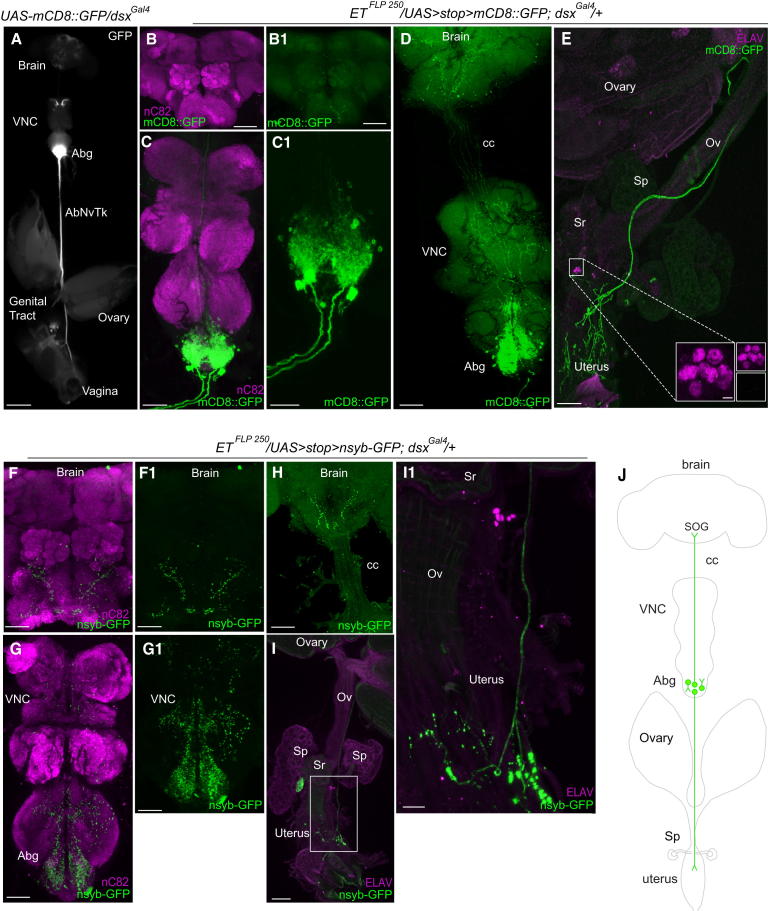
Characterization of Intersected *dsx*-Abg Neurons and Associated Projections (A) Epifluorescence of *dsx^Gal4^*/*UAS-mCD8::GFP* female CNS with AbNvTk innervating the reproductive system. Scale bar represents 200 μm. (B–I) Intersection of *dsx^Gal4^*- neurons and *ET^FLP250^* expressing (B–E) *UAS* > *stop > mCD8::GFP* or (F–I) *UAS* > *stop > nsyb-GFP* in 5-day-old female CNSs and reproductive systems. (B) Female brain lacking *mCD8::GFP* expressing cell bodies, showing (B1) ascending neuronal projections innervating the SOG. (C) Female VNC showing ∼27 *ET^FLP 250^*/*dsx^Gal4^* cell bodies and projections. (C1) Higher magnification of Abg (C). (D–E) Demonstration of *dsx* neuronal projections to (D) the brain and (E) the reproductive system. Inset in (E) shows higher magnification of anterior uterus depicting lack of *mCD8::GFP* expression in *dsx* sensory neurons. (F and G) Presynaptic arborizations of intersecting *dsx* neurons within the (F and FI) posterior SOG and (G and G1) VNC. (H) Cervical connection showing four ascending projections innervating the posterior SOG. (I) Presynaptic arborizations in the internal genitalia, with (I1) a higher resolution of (I) detailing terminalia ramifying on the uterus. (J) Schematic of intersected *dsx*-Abg neurons depicting ascending neurons innervating the central brain, interneurons potentially conveying information to/from central circuits regulating female mating decisions, and descending neurons innervating the uterus. GFP (green); neuropil counterstained with anti-nC82 (magenta); reproductive systems counterstained with anti-ELAV (magenta). Abdominal ganglion (Abg), abdominal nerve trunk (AbNvTk), cervical connective (cc), seminal receptacle (Sr), spermathecae (Sp), and common oviduct (Ov) are indicated. (A–I) Ventral views; anterior, top. (B–I) Scale bars represent 50 μm. Scale bars in inset (E) represent 10 μm.

To map the neurons responsible for this phenotype, we crossed *ET^FLP250^* to the *dsx^Gal4^* driver and the *UAS* > *stop > mCD8::GFP* reporter [28]. mGFP expression was restricted to a small subset of *dsx* neurons within the Abg (27.4 ± 0.5 cells, n = 30; of the ∼300 Abg *dsx* neurons Figures 6C and 6C1), yet no cell bodies were detected in the majority of brain samples (13% ± 4% of them showed one cell stained with GFP, Figures 6B and 6B1), the reproductive system (n = 18) (Figure 6E), and nonneuronal tissues (data not shown). These results show that 27 of the *dsx-*expressing Abg neurons are required for eliciting postmating responses. Intriguingly, we identified approximately four ascending *dsx*-Abg neuronal projections that terminate in the SOG (Figure 6D). Application of the presynaptic reporter *UAS > stop > nsyb-GFP* [28] in combination with *ET^FLP250^* and *dsx^Gal4^* revealed extensive presynaptic arborizations in the posterior SOG, where they probably form synapses (Figures 6F, 6F1, and 6H). These results suggest that *ET^FLP250^*/*dsx* neurons in the Abg convey sensory information, such as stimuli from SP reception, to higher-order centers required for modulation of behavioral and physiological responses to mating.

We detected two descending projections from intersecting neurons in the Abg, which were found to innervate the uterus (Figure 6E), suggesting that these neurons may be involved in directing motor outputs to achieve specific postmating responses, such as oviposition. *nsyb-GFP* expression confirmed presynaptic innervations of Abg-descending *dsx* neurons in the uterus (Figures 6I and 6I1). In addition, we detected a dense region of presynaptic innervations in the Abg (Figures 6G and 6G1), most likely reflecting the presence of localized *dsx* interneurons. From three-dimensional images of mCD8::GFP-labeled intersecting neurons, we could determine the origin of the two descending projections to the reproductive system and two of the four ascending projections to the brain (Figure S5). Given the extensive dendritic arborizations of *ET^FLP250^*/*dsx* neurons within the Abg, it was not possible to identify the exact origin of the two remaining ascending projections.

To evaluate whether *dsx* intersecting neurons were sex specific, we characterized *ET^FLP250^*/*dsx* neurons in the male CNS. Whereas no neurons were found in the majority of brains stained (only 10% of brains showed a single neuron), only four to five *dsx* neurons (n = 20) were found in the male Abg (Figures S6A and S6B). Most males showed one ascending Abg neuronal projection running to the brain, and approximately 45% displayed two descending projections to the reproductive system (Figure S6 and data not shown, respectively). We also detected one to two cells of the male-specific *dsx*-TN1 neuronal cluster [4] in the mesothoracic ganglion, which send projections that terminate in the SOG (Figures S6A and S6C). These results demonstrate clear sexual dimorphism, suggesting a specific role for female *dsx* Abg neurons in regulating sex-specific behaviors.

In summary, we have identified a *doublesex*-derived population of neurons in the Abg, which are critical for postmating responses of *Drosophila melanogaster* females comprising (1) ascending neurons targeting the brain; (2) local interneurons; and (3) descending neurons innervating the reproductive system (see schematic Figure 6J). Our investigations suggest a compelling model whereby female-specific *dsx* neurons play a pivotal role in the coordinated regulation of behavioral and physiological responses after copulation—from reception of stimulatory inputs and transmission of these signals to higher-order centers for processing to generation of the associated behavioral motor outputs.

## Discussion

Our results show that in the female, *dsx* neurons associated with the internal genitalia not only form a component part of the previously described *fru*^+^/*ppk*^+^ network, but in fact define a more minimal SP-responsive neural circuit capable of inducing postmating changes, such as reduced receptivity, increased levels of rejection, and egg deposition.

In addition to these “classic” postmating behavioral responses, we also noted SP signaling to *dsx* neurons induces postmating changes in locomotor activity between unmated and mated females. Studies have shown that *Drosophila* males court immobilized females less than moving females; essentially, males react to changes in female locomotion [20, 29, 30], suggesting a causal link between female locomotion and increased courtship levels. It has been proposed that males are “acoustically tuned” to signals generated by active females, stimulating increased courtship by changing the attention state of the male [21]. Therefore, female mobility appears to contribute to her “sex appeal” and decreased locomotion in mated females is likely to affect the male's willingness to copulate.

### SP-Responsive Sensory Neurons Regulate Different Aspects of the Postmating Response

The female's nervous system must have the capacity to receive, and interpret, postcopulatory signals derived from the male seminal package to direct physiological and behavioral responses required for successful deposition of fertilized eggs. We demonstrated that two *dsx* clusters, composed of three bilateral neurons of the uterus, comprise a more defined component of the SP-responsive sensory circuit. In addition, we have shown the majority of other *dsx* neurons originating on the internal genitalia to coexpress *ppk*. As *ppk* neurons are mechanosensory [16, 17], these may be acting as uterine stretch receptors, facilitating sperm and egg transport, fertilization, and oviposition [14]. Silencing neural function of *ppk* neurons appears to inhibit egg deposition, presumably by impeding egg transport along the oviducts [15]. Similarly, in *dsx^Gal4^* females expressing TNT no egg deposition is ever observed, with unfertilized eggs atrophying in the lateral oviducts [4]. In contrast, when *fru*^+^ neurons are silenced, deposition of successfully fertilized eggs is still observed [15], suggesting that different subsets of the *dsx*^+^/*fru*^+^/*ppk*^+^ SP-responsive sensory circuit may direct distinct postmating behavioral responses. As SP has been detected in the hemolymph of mated females [31], it has been suggested that this peptide could pass from the reproductive tract into the hemolymph to reach CNS targets [9]. The fact that neither receptivity nor oviposition was restored to control levels when *ppk*-*Gal80* (or *Cha-Gal80*) was expressed in *dsx^Gal4^*/*UAS-mSP* flies opens the possibility that SP expression might affect additional *dsx* neurons in the CNS.

### Coexpression of SPR and *dsx* in the Female Reproductive Organs and Potential Functions in Directing Postmating Responses

Triggering of postmating responses via SP reception appears to occur via a small number of neurons expressing SPR on the female reproductive tract [14, 15]; however, SPR is also found on surface regions of the CNS as well as in endocrine glands and other reproductive tissues [13]. Surprisingly, SPR may even be detected in the *Drosophila* male CNS, where no exposure to SP would be expected, and in insects that apparently lack SP-like peptides [13, 32]. SPRs are therefore potentially responsive to other ligands [32], performing functions other than those associated with postmating responses in the diverse tissues in which SPRs are expressed.

We found extensive coexpression of *dsx*-expressing cells and SPR in the epithelium of the lower oviduct and spermathecae in females (Figure S3). However, mSP expression (or SPR downregulation) specifically in spermathecal secretory cells (SSC) [33] or oviduct epithelium cells [34] had no effect on receptivity or egg laying (data not shown). In agreement with our rescue experiments using neuronal Gal80 drivers to intersect Gal4-responsive UAS expression in *dsx* cells, this suggests that these cells are neither neuronal nor directly involved in SP-mediated postmating behaviors (Figures 1, 2, and 3). SPR staining in the CNS was more difficult to determine given the limitations of the antibody; while no colocalization in the brain was observed, apparent coexpression was observed between SPR and a small subset of ventral *dsx*-Abg neurons (Figure S3).

### How Does the Female's CNS Integrate Information about Reproductive Status to Switch Behavior and Physiology Postcopulation?

Our results indicate that *dsx*-Abg neurons are required for the induction and regulation of specific components of the postmating response. It has been shown that inhibition of neurotransmission in *apterous*-expressing Abg neurons impairs SP-mediated postmating changes in receptivity and oviposition, emphasizing the importance of these neurons in the modulation of postmating responses [35].

The level of *dsx* neuronal expression within the Abg and their associated fascicles projecting to the brain, where they form extensive presynaptic arborizations within the SOG, coupled with the effects that impairment of function in these neurons has on postmating responses, speaks to the involvement of these neurons in relaying information from the reproductive tract to the brain (Figure 6). That *dsx*-Abg neurons also project, and form presynaptic arborizations on the uterus, and that the effects on postmating responses when their function is impaired again argue that these neurons play a direct role in mediating processes such as egg fertilization and oviposition. Interestingly, most *dsx* intersecting neurons are specific to females (Figures 6 and S6). Sex-specific behaviors can arise from either shared circuits between males and females that operate differently and/or sex-specific circuits that result from the presence/absence of unique circuit components in one sex versus the other. Our results support the latter.

The VNC has been implicated in the modulation of postmating responses, with an identified focus specifically involved in ovulation and transfer of eggs into the uterus for fertilization [35]. Octopaminergic modulatory neurons located at the distal tip of the VNC projecting to the reproductive tract are required for triggering ovulation, possibly by regulating muscle contractions in the ovaries and oviducts [36, 37]. Since ablation of the pars intercerebralis revealed an additional focus for egg laying in the head [38], and the brain appears to be required for sexual behaviors, such that decapitated virgin females neither mate nor lay eggs [39], it seems likely that neurons in the Abg also require signals from the brain to regulate postmating responses such as egg transport, fertilization, and deposition.

### Integrating Sensory Inputs

Higher-order circuits in the female brain must be capable of integrating sensory inputs from the olfactory, auditory, and reproductive systems to decide between the alternative actions of acceptance or rejection of the male. Early gynandromorph studies mapped a region of the dorsal brain that must be female for an animal to be receptive [40]; it has been recently shown that the majority of *dsx* neuronal clusters are located in this region [4]. While neurons coexpressing *dsx* and *fru* in male brains define a more restricted circuitry for determining male mating decisions, in females no overlap between *dsx^+^* and *fru^+^* neurons is observable in the brain (data not shown). It is also important to note that the sex-specific Fru isoform is absent in females; thus any circuits that are actively specified in the female are likely to depend on the female isoform Dsx^F^. Most *dsx* neurons in the brain are found in the lateral protocerebrum, a region where multiple sensory inputs are thought to be integrated and discrete motor actions selected and coordinated. Further high-resolution functional and connectivity mapping will help to define which neurons participate in specific pre- and postmating behaviors in the female, allowing us to integrate circuit architecture with underlying cellular and synaptic properties. Future experiments will define what activity patterns trigger these behaviors and what activity patterns correlate with these behaviors.

## Supplemental Information

Document S1. Supplemental Experimental Procedures, Figures S1–S6, and Table S1

## References

[1] Villella A., Hall J.C. (2008). Neurogenetics of courtship and mating in Drosophila. Adv. Genet..

[2] Rideout E.J., Billeter J.C., Goodwin S.F. (2007). The sex-determination genes fruitless and doublesex specify a neural substrate required for courtship song. Curr. Biol..

[3] Kimura K., Hachiya T., Koganezawa M., Tazawa T., Yamamoto D. (2008). Fruitless and doublesex coordinate to generate male-specific neurons that can initiate courtship. Neuron.

[4] Rideout E.J., Dornan A.J., Neville M.C., Eadie S., Goodwin S.F. (2010). Control of sexual differentiation and behavior by the doublesex gene in Drosophila melanogaster. Nat. Neurosci..

[5] von Philipsborn A.C., Liu T., Yu J.Y., Masser C., Bidaye S.S., Dickson B.J. (2011). Neuronal control of Drosophila courtship song. Neuron.

[6] Connolly K., Cook R. (1973). Rejection responses by female Drosophila melanogaster: their ontogeny, causality and effects upon the behaviour of the courting male. Behaviour.

[7] Ejima A., Nakayama S., Aigaki T. (2001). Phenotypic association of spontaneous ovulation and sexual receptivity in virgin females of Drosophila melanogaster mutants. Behav. Genet..

[8] Spieth H.T., Ringo J.M., Ashburner M., Carson H.L., Thompson J.N. (1983). Mating behavior and sexual isolation in Drosophila.

[9] Kubli E. (2003). Sex-peptides: seminal peptides of the Drosophila male. Cell. Mol. Life Sci..

[10] Liu H., Kubli E. (2003). Sex-peptide is the molecular basis of the sperm effect in Drosophila melanogaster. Proc. Natl. Acad. Sci. USA.

[11] Chapman T., Bangham J., Vinti G., Seifried B., Lung O., Wolfner M.F., Smith H.K., Partridge L. (2003). The sex peptide of Drosophila melanogaster: female post-mating responses analyzed by using RNA interference. Proc. Natl. Acad. Sci. USA.

[12] Chen P.S., Stumm-Zollinger E., Aigaki T., Balmer J., Bienz M., Böhlen P. (1988). A male accessory gland peptide that regulates reproductive behavior of female D. melanogaster. Cell.

[13] Yapici N., Kim Y.J., Ribeiro C., Dickson B.J. (2008). A receptor that mediates the post-mating switch in Drosophila reproductive behaviour. Nature.

[14] Häsemeyer M., Yapici N., Heberlein U., Dickson B.J. (2009). Sensory neurons in the Drosophila genital tract regulate female reproductive behavior. Neuron.

[15] Yang C.H., Rumpf S., Xiang Y., Gordon M.D., Song W., Jan L.Y., Jan Y.N. (2009). Control of the postmating behavioral switch in Drosophila females by internal sensory neurons. Neuron.

[16] Grueber W.B., Ye B., Moore A.W., Jan L.Y., Jan Y.N. (2003). Dendrites of distinct classes of Drosophila sensory neurons show different capacities for homotypic repulsion. Curr. Biol..

[17] Adams C.M., Anderson M.G., Motto D.G., Price M.P., Johnson W.A., Welsh M.J. (1998). Ripped pocket and pickpocket, novel Drosophila DEG/ENaC subunits expressed in early development and in mechanosensory neurons. J. Cell Biol..

[18] Nakayama S., Kaiser K., Aigaki T. (1997). Ectopic expression of sex-peptide in a variety of tissues in Drosophila females using the P[GAL4] enhancer-trap system. Mol. Gen. Genet..

[19] Tompkins L., Gross A.C., Hall J.C., Gailey D.A., Siegel R.W. (1982). The role of female movement in the sexual behavior of Drosophila melanogaster. Behav. Genet..

[20] Siegel R.W., Hall J.C. (1979). Conditioned responses in courtship behavior of normal and mutant Drosophila. Proc. Natl. Acad. Sci. USA.

[21] Ejima A., Griffith L.C. (2008). Courtship initiation is stimulated by acoustic signals in Drosophila melanogaster. PLoS ONE.

[22] Rubinstein C.D., Rivlin P.K., Hoy R.R. (2010). Genetic feminization of the thoracic nervous system disrupts courtship song in male Drosophila melanogaster. J. Neurogenet..

[23] Lai S.L., Lee T. (2006). Genetic mosaic with dual binary transcriptional systems in Drosophila. Nat. Neurosci..

[24] Mellert D.J., Knapp J.M., Manoli D.S., Meissner G.W., Baker B.S. (2010). Midline crossing by gustatory receptor neuron axons is regulated by fruitless, doublesex and the Roundabout receptors. Development.

[25] Salvaterra P.M., Kitamoto T. (2001). Drosophila cholinergic neurons and processes visualized with Gal4/UAS-GFP. Brain Res. Gene Expr. Patterns.

[26] Bohm R.A., Welch W.P., Goodnight L.K., Cox L.W., Henry L.G., Gunter T.C., Bao H., Zhang B. (2010). A genetic mosaic approach for neural circuit mapping in Drosophila. Proc. Natl. Acad. Sci. USA.

[27] Hamada F.N., Rosenzweig M., Kang K., Pulver S.R., Ghezzi A., Jegla T.J., Garrity P.A. (2008). An internal thermal sensor controlling temperature preference in Drosophila. Nature.

[28] Yu J.Y., Kanai M.I., Demir E., Jefferis G.S., Dickson B.J. (2010). Cellular organization of the neural circuit that drives Drosophila courtship behavior. Curr. Biol..

[29] Hall J.C. (1978). Courtship among males due to a male-sterile mutation in Drosophila melanogaster. Behav. Genet..

[30] Streisinger G. (1948). Experiments on sexual isolation in Drosophila. IX. Behavior of males with etherized females. Evolution.

[31] Pilpel N., Nezer I., Applebaum S.W., Heifetz Y. (2008). Mating-increases trypsin in female Drosophila hemolymph. Insect Biochem. Mol. Biol..

[32] Kim Y.J., Bartalska K., Audsley N., Yamanaka N., Yapici N., Lee J.Y., Kim Y.C., Markovic M., Isaac E., Tanaka Y., Dickson B.J. (2010). MIPs are ancestral ligands for the sex peptide receptor. Proc. Natl. Acad. Sci. USA.

[33] Schnakenberg S.L., Matias W.R., Siegal M.L. (2011). Sperm-storage defects and live birth in Drosophila females lacking spermathecal secretory cells. PLoS Biol..

[34] Lee H.G., Rohila S., Han K.A. (2009). The octopamine receptor OAMB mediates ovulation via Ca2+/calmodulin-dependent protein kinase II in the Drosophila oviduct epithelium. PLoS ONE.

[35] Soller M., Haussmann I.U., Hollmann M., Choffat Y., White K., Kubli E., Schäfer M.A. (2006). Sex-peptide-regulated female sexual behavior requires a subset of ascending ventral nerve cord neurons. Curr. Biol..

[36] Monastirioti M. (2003). Distinct octopamine cell population residing in the CNS abdominal ganglion controls ovulation in Drosophila melanogaster. Dev. Biol..

[37] Rodríguez-Valentín R., López-González I., Jorquera R., Labarca P., Zurita M., Reynaud E. (2006). Oviduct contraction in Drosophila is modulated by a neural network that is both, octopaminergic and glutamatergic. J. Cell. Physiol..

[38] Boulétreau-Merle J. (1976). [Destruction of the pars intercerebralis in drosophila melanogaster: effect on the fecundity and the stimulation through copulation]. J. Insect Physiol..

[39] Ejima A., Smith B.P., Lucas C., Levine J.D., Griffith L.C. (2005). Sequential learning of pheromonal cues modulates memory consolidation in trainer-specific associative courtship conditioning. Curr. Biol..

[40] Szabad J., Fajszi C. (1982). Control of female reproduction in Drosophila: genetic dissection using gynandromorphs. Genetics.

