# Supplementary material for: Neural Circuitry Underlying Drosophila Female Postmating Behavioral Responses
Source: Curr Biol. 2012 Jul 10;22(13):1155–65. doi: 10.1016/j.cub.2012.04.062 (PMC3396843; doi:10.1016/j.cub.2012.04.062)
Supplement: Document S1. Supplemental Experimental Procedures, Figures S1–S6, and Table S1 [file mmc1.pdf]

Supplemental Information

Current Biology, Volume *22*

Neural Circuitry Underlying *Drosophila* Female Postmating Behavioral Responses

Carolina Rezával, Hania J. Pavlou, Anthony J. Dornan, Yick-Bun Chan, Edward A. Kravitz, and Stephen F. Goodwin

Supplemental Experimental Procedures

D. melanogaster strains and crosses

All flies were raised at 25°C on standard medium in a 12 hr light /12 hr dark cycle. Wild-type flies were obtained from a *Canton-S* strain*.* We obtained the *UAS-pStingerII*, *UAS-mCD8::GFP*, *UAS-redStinger/CyO, Cha-Gal4, Tdc2-Gal4; glu-Gal4; and UAS-dcr2* lines from Bloomington *Drosophila* Stock Center. Other fly stocks used were *Cha-Gal80* [S1], *th-Gal4* [S2], dsx^{Gal4} [S3], *trh-Gal4* [S4], *gad1-Gal4* [S5]. *elav-Gal80* and $UAS\text{-}TNT_G$ (provided by S. Sweeney), *UAS>stop>mCD8::GFP*, *UAS>stop>TNT*, $UAS\text{>}stop\text{>}trpA1^{mCherry}$, *UAS-SPR-IR1*, w^+; *Df(1)Exel6234 (a.k.a Df(1)SPR)*, *UAS-SPR* and *UAS>stop>nsyb-GFP* (provided by B. Dickson), *ppk-Gal80, UAS-mSP;;UAS-mSP* (provided by Yuh-Nung Jan); *ppk-Gal4* and *ppk-eGFP* (provided by W.B Grueber), $lexAop\text{-}tdTomato::nls;fru^{P1LexA}$ (provided by B. Baker, Janelia Farm), *nsyb-Gal80, gat1-Gal4* (provided by J. Simpson, Janelia Farm). SPR RNAi (*UAS-SPR-IR*) was used with *UAS-dcr2* on the first chromosome. Y-B Chan and EA Kravitz (Harvard Medical School) generated the collection of ET^{FLP} lines. Briefly, an enhancer trap FLP insertion on chromosome I (provided by Liqun Luo) was mobilized onto the autosome by crossing it to transposase line y^-,w^-*; Δ2-3*. A total of ~350 ET^{FLP} lines were generated and balanced with either *CyO* or *TM3*. Individual

enhancer trap FLP lines were then crossed to *elav-Gal4*; *UAS>stop> mCD8::GFP* to assess expression patterns. 1-3 day old flies were dissected and immunostained to check for consistent GFP expression in the nervous system (YB Chan and EA Kravitz, unpublished). The use of an independent *dsx* GAL4 driver line, $dsx^{[Gal4\ 1]}$ (provided by B. Baker), led to similar neuroanatomical results to that observed in ET^{FLP250}*/UAS>stop>mCD8::GFP;* dsx^{Gal4} females.

Immunohistochemistry

Flies were reared at 25°C and aged for 4–6 days (for CNS) or for 7-10 days (for reproductive system) prior to dissection and staining as previously described [S6]. Primary antibodies used were: rabbit anti-SPR (1:500 for CNS or 1:2000 for the reproductive system, provided by B. Dickson), rabbit anti-GFP (1:1000, Invitrogen Molecular Probes, Carlsbad, CA), chicken anti-GFP (1:1500, Abcam), mouse mAb nC82 (1:10, DSHB, Univ. of Iowa, IA), and rat anti-ELAV (1:500, DSHB, Univ. of Iowa, IA). Secondary antibodies used were: anti-rat Alexa Fluor488, anti-rabbit Alexa Fluor488, and anti-mouse Alexa Fluor546 conjugates (1:300 Invitrogen Molecular Probes, Carlsbad, CA) and anti-chicken Alexa Fluor488 (1:500 Invitrogen Molecular Probes, Carlsbad, CA). Confocal stacks were taken with a Leica SP5 with 10 or 20 x /0.7 NA dry objectives. Zeiss LSM 710 Meta confocal was used to take 40X images. Epifluorescence microscopy on whole-mount preparations was performed on a Zeiss SteREO Lumar V.12 Stereomicroscope and captured via a ZeissAxiocam. Images were processed in Amira 5.2 (Mercury Systems). In some cases, visualization was limited to a subset of confocal sections to permit a clear projection of the structure in question.

Behavioral assays

All behavioral experiments were carried out at 25°C. *Receptivity*: Individual 3-5 day old virgin females were introduced into a vial with yeast containing two naïve Canton-S males, the percent of females achieving copulation within 1 hr was measured. *Remating*: receptivity was again measured using the same mated females 48 hr after copulation. *Egg-laying*: individual 4–5 day old females were transferred to a fresh vial with media and allowed to lay eggs for 48 hr at 25°C. To score courtship, locomotion and rejection parameters (Figures 1-3), individual females were introduced into a round chamber (19 mm diameter × 4 mm height) with an individual naïve CS male. The following parameters were measured during a 3 min observation period (or until mating occurred) starting from courtship initiation (first bout lasting over 3 seconds): *Movement during courtship*: percentage of time females spent moving while being actively courted by the male, *Movement in the absence of active courtship*: percentage of time females spent moving when male was not actively courting, *Ovipositor extrusion*: number of ovipositor extrusion performed by the female per minute during the observation period of courtship and *Male courtship index*: percentage of time during which a male displays any of the courtship steps, including following, tapping, wing extension, licking, and attempted copulation.

Neuronal Silencing experiments

For *UAS-TNT* experiments, behavior was measured as previously described [3]. Individual females were introduced into a round courtship chamber (19 mm diameter × 4 mm height) with an individual naïve *Canton-S* male. O*vipositor extrusion* was measured during a 3 min observation period (or until mating occurred) starting from the time of courtship initiation. *Male courtship index* was measured during a 10-min observation period starting from courtship initiation. Remating was assessed 24 hr

after the first copulation. Courtship latency is the time from when males are introduced to females until the initiation of courtship (first bout of courtship lasting over 3 seconds).

Thermal activation experiments

Individual females were introduced into a round courtship chamber (10 mm diameter × 4 mm height) with an individual naïve *Canton-S* male, which was then placed on a heating plate (Techne Dri-block heater). Experiments were performed either at 22°C or 31°C. *Receptivity* was measured as percent of females achieving copulation within 1 hr. *Ovipositor extrusion* was measured during a 3 min observation period (or until mating occurred) starting from the initiation of courtship. Male courtship index was measured during a 10-min observation period from courtship initiation. Courtship latency is the time from when males are introduced to females until the initiation of courtship (first bout of courtship lasting over 3 seconds).

Experiments with decapitated female targets

Male courtship towards decapitated female targets was measured in individual chambers of 12-well polystyrene plates (each chamber dimension is 10 mm diameter × 5 mm height) during a 10-min observation period (or until copulation) from the time of courtship initiation. 'Headless' females were placed in the centre of the chamber ~30 min before transferring an individual naïve *Canton-S* male.

Statistics

Behavioral means were compared using Kruskal-Wallis ANOVA test and Dunn's post hoc statistical test where indicated. For Fisher's exact test, two-tail *p* values were

compared with controls. Statistical analyses were performed with the Prism software (version 5.0b, SPSS Inc.).

Supplemental Figures

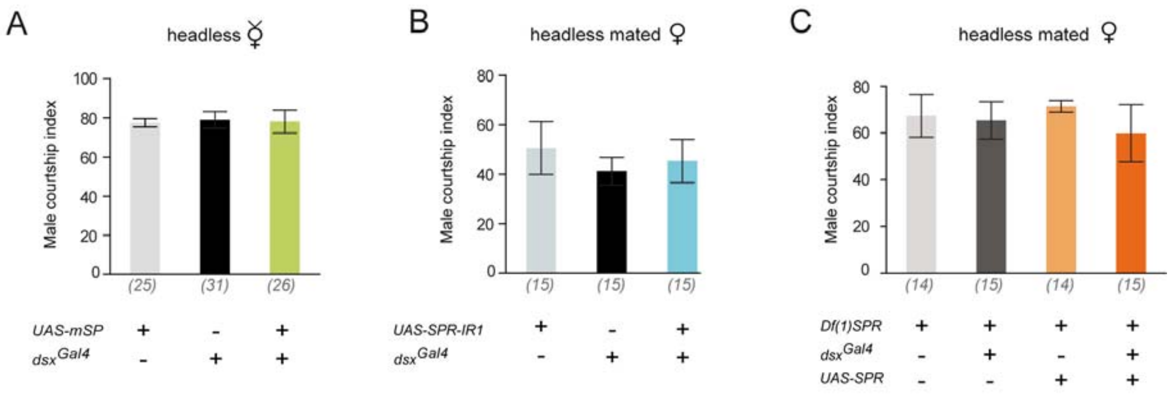

Figure S1. Male courtship index in the absence of female behavioral cues.

Male courtship index of naïve wild-type males paired with (A) decapitated virgin females and (B-C) decapitated mated females (Kruskal-Wallis ANOVA test). No significant differences were found between the genotypes of each experimental cohort. Error bars indicate SEM. Genotypes indicate virgin or mated females, n values shown in parentheses.

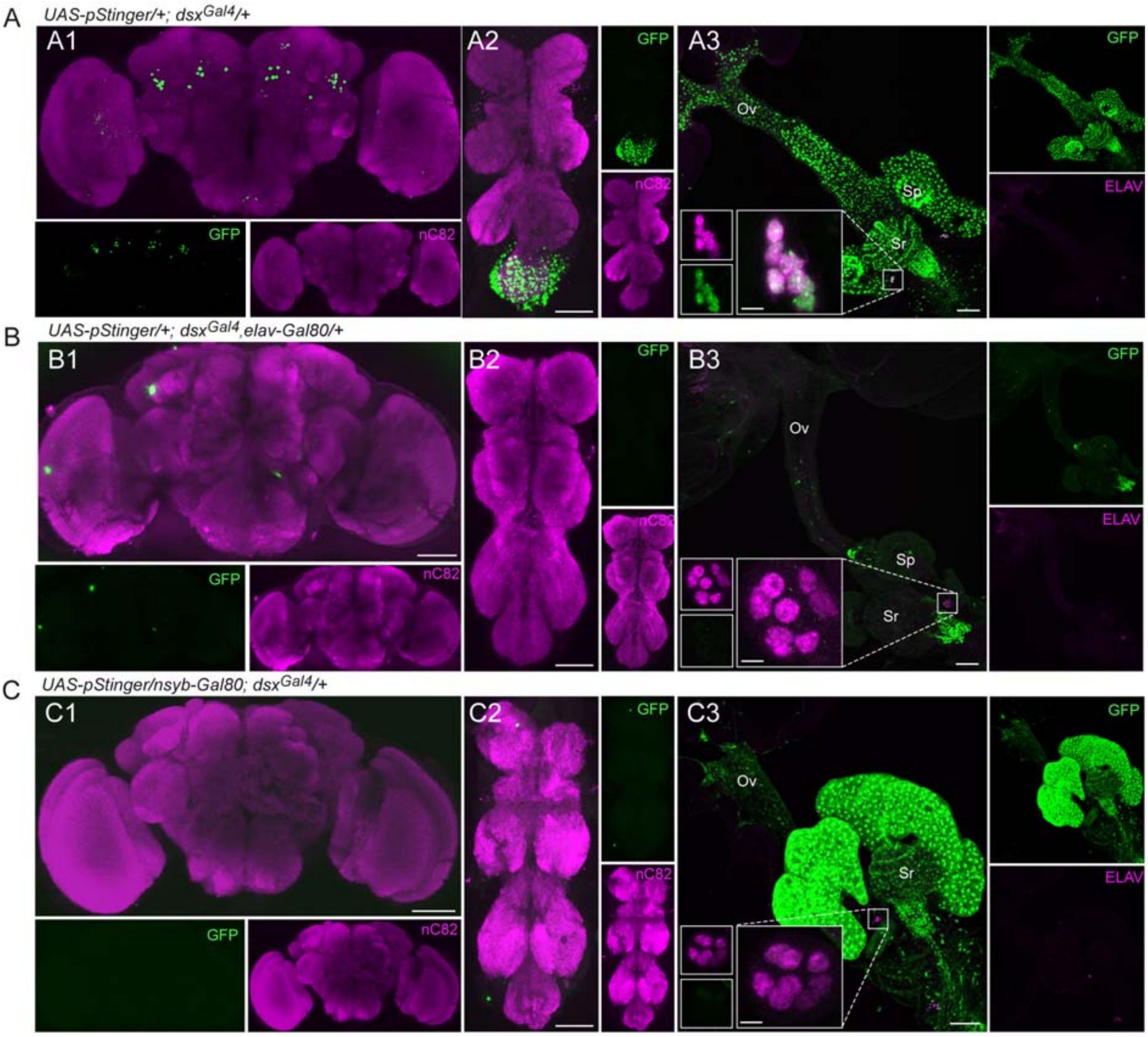

Figure S2. Differential effects of neuronal Gal4 inhibitors, *elav-Gal80* and *syb-Gal80*, on *dsx* expression.

(A) Expression of nGFP (green) in *UAS-pStinger/+; dsx*^{Gal4}/+ 5 day-old female (A1) brain, (A2) VNC and (A3) reproductive system. Inset in (A3) shows co-localization between anti-ELAV (magenta) and *dsx* (green). (B1-3) Expression of nGFP (green) in *UAS-pStinger/+; dsx*^{Gal4}/+ 5 day-old female, also expressing the neuronal Gal4 repressor *elav-Gal80.* No expression of GFP is detected in any *dsx*^{Gal4}-neuronal clusters in the (B1) brain and (B2) VNC. (B3) Ectopic repression of nGFP in non-neuronal *dsx*^{Gal4}-expressing cells is observed within the lateral and common oviduct

(Ov), spermathecae (Sp), seminal receptacle (Sr) and uterus. Inset in (B3) shows absence of nGFP in neurons (ELAV-positive cells, magenta). (C1-3) Expression of nGFP (green) in *UAS-pStinger/+; dsx*^{Gal4}/+ 5 day-old female, also expressing the neuronal Gal4 repressor *nsyb-Gal80.* No expression of nGFP is detected in any *dsx*^{Gal4}-neuronal clusters within the (C1) brain and (C2) VNC. (C3) No apparent ectopic repression of non-neuronal *dsx*^{Gal4}-expressing cells is detected within the internal reproductive system. Inset in (C3) shows absence of nGFP in neurons (ELAV-positive cells, magenta). nGFP expression is shown in green and neuropil was counterstained with anti-nC82 (magenta). Reproductive systems were counterstained with anti-ELAV (magenta). Ventral views; anterior, top. Scale bars = 50 μm. Scale bars in insets = 10 μm.

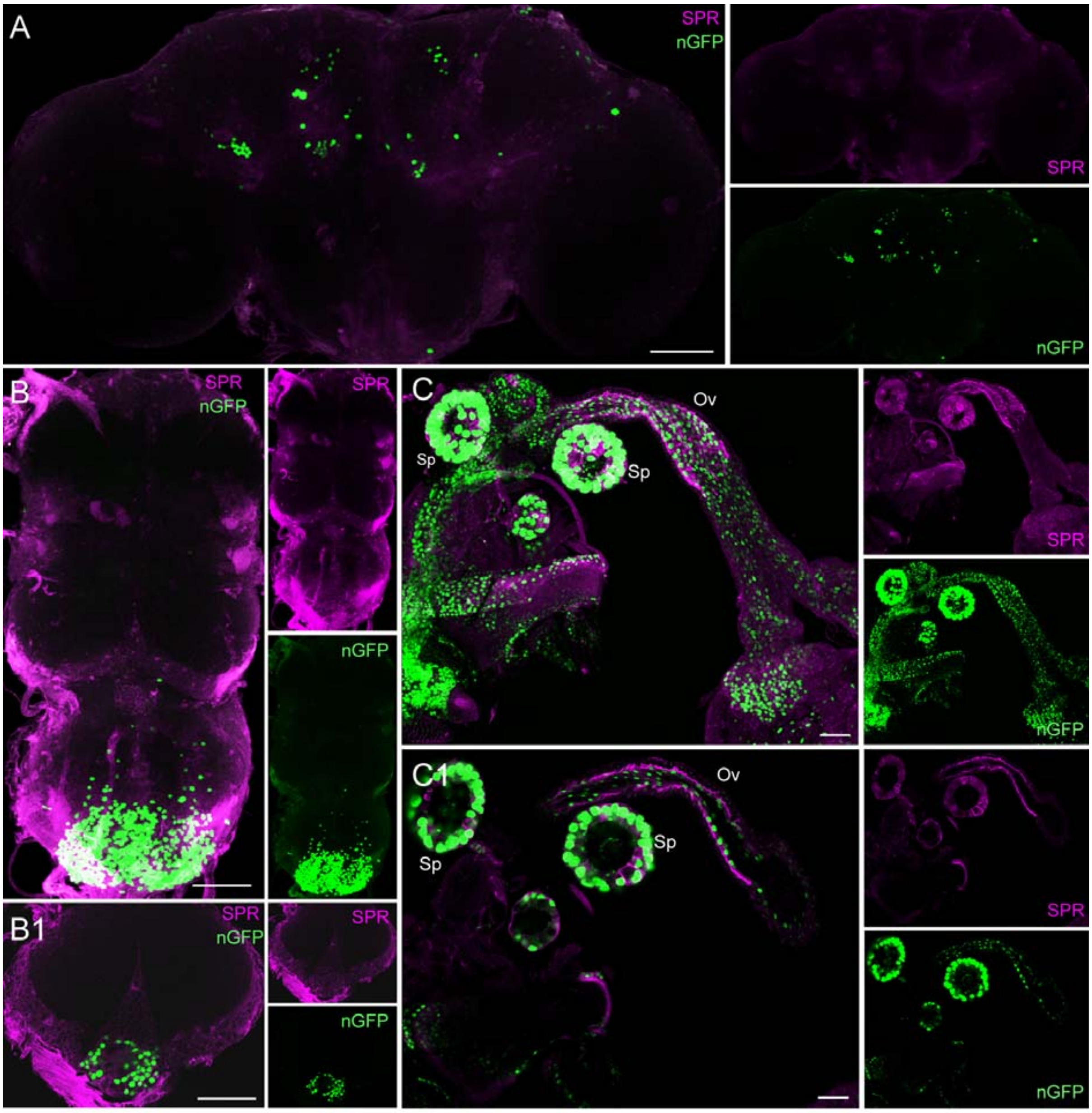

Figure S3. ***dsx*^{Gal4} and SPR expression in the CNS and internal genitalia of 5-day-old adult females.**

(A-B) *UAS-pStinger/+; dsx*^{Gal4}/+ female CNS co-stained with anti-SPR. (A) No apparent co-localization detected in the brain; however, (B) a small subset of *dsx*^{Gal4}-expressing neurons of the Abg in the VNC appears to express SPR on their membrane. (B1) Subset of confocal sections of the VNC (B) appear to show expression of SPR on the membrane of *dsx* neuronal cell bodies in the dorsal Abg. (A-B) ventral views; anterior, up. (C) Reproductive tract of *UAS-pStinger*/+; *dsx*^{Gal4}/+

females stained with anti-SPR. Co-localization between *dsx*^{Gal4} and SPR occur within the lower common oviduct (Ov) and spermathecae (Sp). (C1) Subset of confocal sections detailing co-expression of SPR and *dsx* within the structures indicated. Anti-SPR (magenta) and nGFP (green). Scale bars = 50 μm.

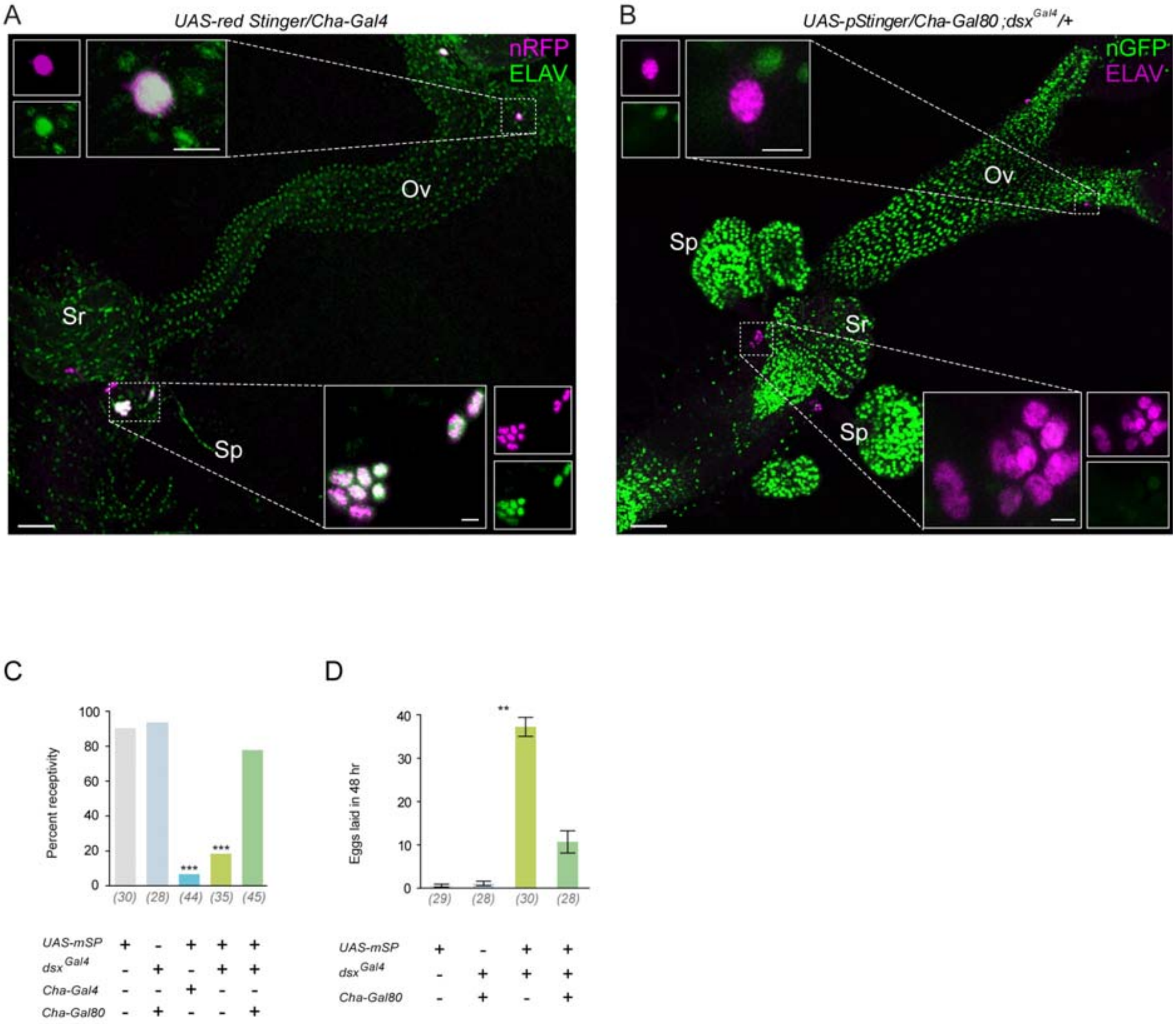

Figure S4. *dsx* sensory neurons in the female reproductive system appear cholinergic.

(A) Sensory neurons in the adult female reproductive system are positive for *Cha-Gal4*. Pan-neuronal antibody, anti-ELAV (green), is shown to co-localize with *Cha-Gal4*/*UAS-redStinger* cells (nRFP; magenta). Insets in (A) show clear overlap between *Cha-Gal4* expressing cells and ELAV in the lateral oviduct and uterus, at higher magnification. (B) *Cha-Gal80* represses nGFP expression in *dsx* neurons of the genital tract. *UAS-pStinger*; *dsx*^{Gal4} (nGFP, green) female reproductive system also expressing *Cha-Gal80* co-stained with anti-ELAV (magenta). Insets in (B) clearly demonstrate repression of nGFP in *dsx*-expressing neurons in the lateral oviduct and

uterus, at higher magnification. Scale bars = 50 µm in (A) and (B), and 10 µm in insets. Seminal receptacle (Sr), spermathecae (Sp), common oviduct (Ov) indicated. (C-D) *Cha-Gal80* expression reduces postmating behaviors in *UAS-mSP/*+;;*dsx*^{Gal4}/*UAS-mSP* virgin females. (C) Percentage receptivity (***p<0.0001, Fisher exact test). (D) Egg-laying (***p<0.0001, Kruskal-Wallis ANOVA test). Error bars indicate SEM. Genotypes indicate virgin females. Target males were wild-type. n values shown in parentheses.

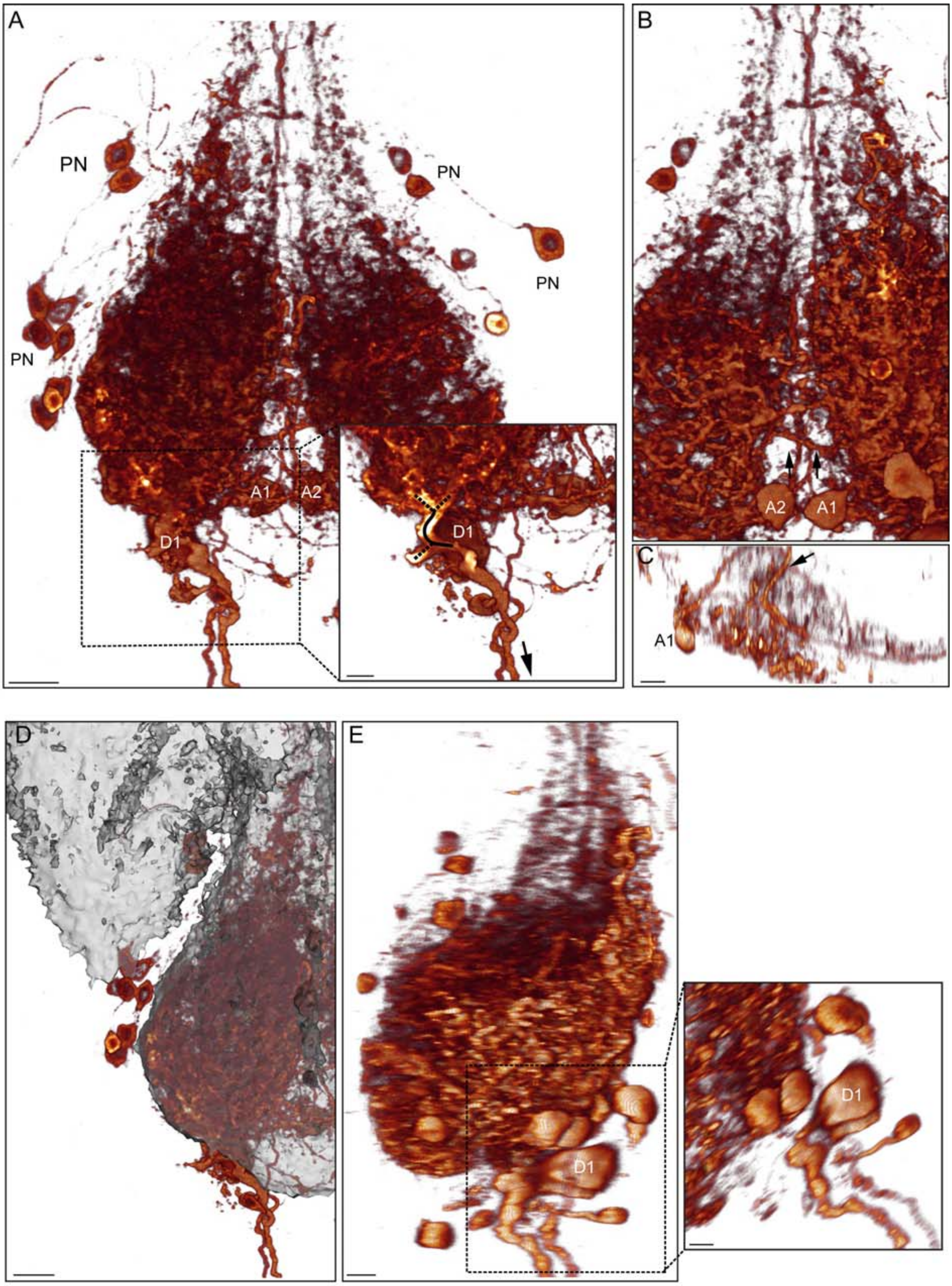

Figure S5. Three-dimensional images of a female Abg showing *ET$^{FLP\ 250}$/dsx^{Gal4}* neurons.

(A-D) Voltex rendered images of female Abgs illustrating neural topography of $ET^{FLP\ 250}/dsx^{Gal4}$ intersected neurons and their associated neurites. (A) Abg (ventral view) showing $ET^{FLP\ 250}/dsx^{Gal4}$ soma and associated extensive dendritic arborizations. A pair of centrally located dorsal neurons, A1 and A2, give rise to ascending projections terminating in the dorsal SOG. Additional ascending projections are likely to originate from peripheral neurons (PN) indicated. D1 represents one of the bilaterally paired somas contributing to descending projections terminating on the uterus. A subset of confocal sections (inset of A) represents a magnified detail of Abg highlighting the D1 neuron. The D1 soma gives rise to a descending axonal projection, which ramifies on the internal genitalia (arrow), as well as extensive dendritic arborization, formed from three branches (dashed lines) arising from a primary dendritic stem, within the Abg itself. (B) Midline of the Abg (dorsal view) showing A1 and A2 somas and their associated ascending projections (arrows) that ultimately target the brain. (C) A subset of confocal sections of the Abg (lateral view) highlights the A1 neuron (A2 out of plane of view). The A1 axon forms a characteristic dorsal loop (arrow) within in the Abg before extending to the anterior VNC. (D) Left half female Abg (ventral, coronal view) showing intersecting neurons (orange) and nc82 neuropil marker (grey). (E) Detail of left half female Abg (ventral, coronal view) highlighting descending projections of the D1 neuron. Inset in (D) is a magnified and rotated medio-lateral view of the D1 neuron and its descending axon and dendritic projections. Scale bars = 10 μm.

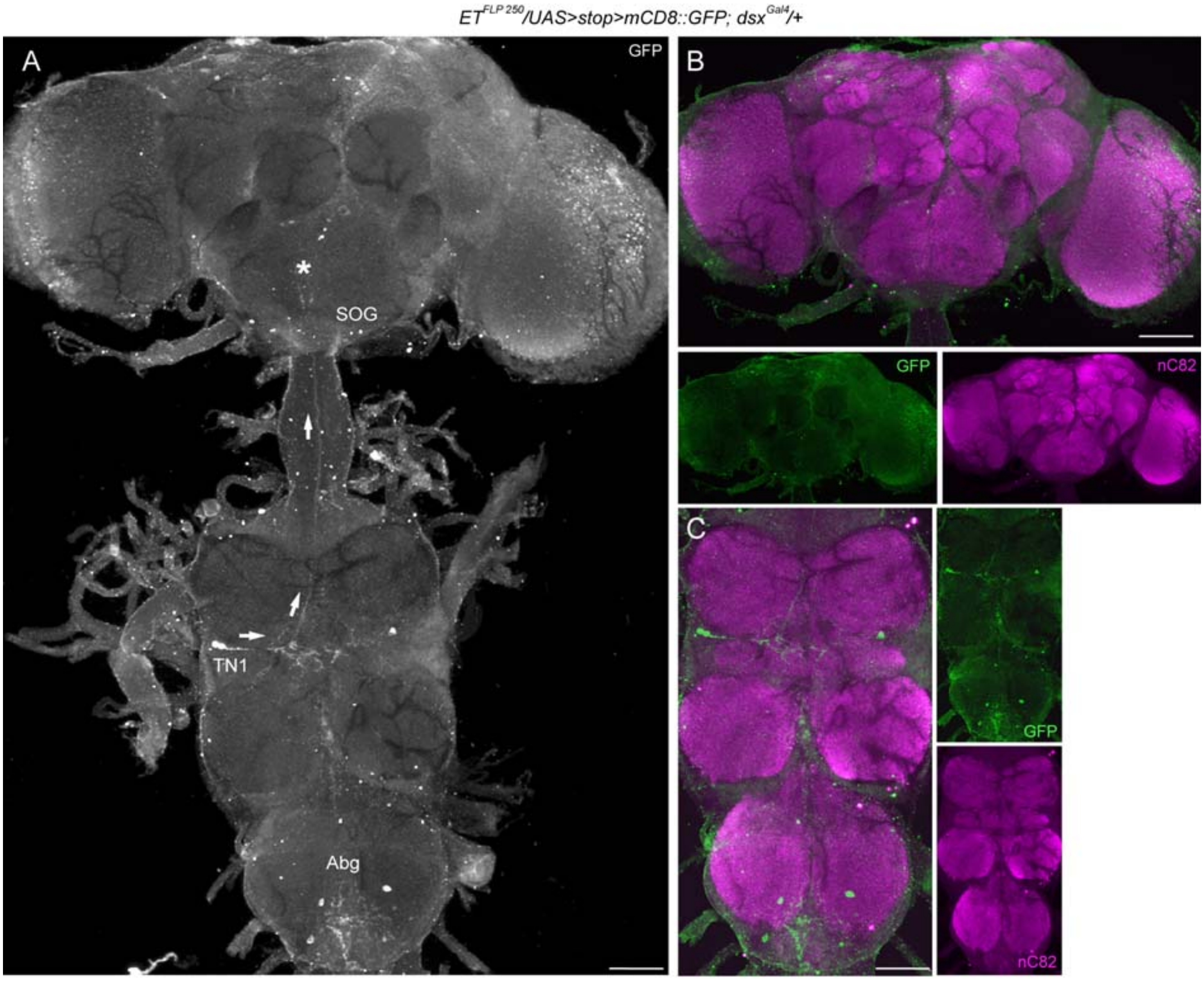

Figure S6. Membrane-bound GFP expression in *$ET^{FLP\ 250}$/dsx^{Gal4}* intersected neurons, and associated projections, in males.

(A-C) Intersection of *dsx^{Gal4}*-expressing neurons using *ET^{FLP250}* and *UAS>stop>mCD8::GFP* (green) in a 5 day old male CNS. (A) Male CNS exhibiting expression in *dsx* neurons in the VNC sending projections to innervate the posterior SOG. Note the male-specific *dsx*-TN1 neuronal cluster in the mesothoracic ganglion that sends projections through the cervical connective (arrows) terminating within the posterior SOG (asterisk). (B) Detail of brain from (A) demonstrating the absence of *mCD8::GFP* expressing neurons. (C) Detail of VNC from (A) exhibiting bilateral expression in TN1 neurons in the mesothoracic ganglion, in addition to 4 neurons in the Abg, with their respective projections. Neuropil counterstained with anti-nC82 (magenta). Ventral views; anterior, top. Scale bars = 50 μm.

Genotype	Receptive (%)	n	Gal4 Driver Description
UAS-mSP/+;; dsx^{Gal4}/UAS-mSP	18.4 ± 3.8	37	*doublesex* neurons
UAS-mSP/+;;th-Gal4/UAS-mSP	95.0 ± 5.7	32	Dopaminergic neurons
UAS-mSP/+;Tdc2 -Gal4/+;UAS-mSP/+	91.7 ± 4.1	30	Octopaminergic neurons
UAS-mSP/+;gad1-Gal4/+;UAS-mSP/+	80.9 ± 3.2	30	GABAergic neurons
UAS-mSP/+;gat1-Gal4/+;UAS-mSP/+	92.5 ± 3.8	30	GABAergic neurons
UAS-mSP/+;glu-Gal4/+; UAS-mSP/+	88.6 ± 5.9	34	Glutamatergic neurons
UAS-mSP/+;;trh-Gal4/UAS-mSP	96.7 ± 3.3	28	Serotonergic neurons
UAS-mSP/+;Cha-Gal4/+;UAS-mSP/+	6.6 ± 2.3	45	Cholinergic neurons

Table S1.

Effects of expressing Gal4 responsive transgenic *UAS-mSP* using variant Gal4 driver lines on virgin female receptivity. n=number of animals tested.

Supplemental references

S1. Kitamoto, T. (2002). Conditional disruption of synaptic transmission induces male-male courtship behavior in Drosophila. Proc Natl Acad Sci U S A *99*, 13232-13237.

S2. Friggi-Grelin, F., Coulom, H., Meller, M., Gomez, D., Hirsh, J., and Birman, S. (2003). Targeted gene expression in Drosophila dopaminergic cells using regulatory sequences from tyrosine hydroxylase. J Neurobiol *54*, 618-627.

S3. Rideout, E.J., Dornan, A.J., Neville, M.C., Eadie, S., and Goodwin, S.F. (2010). Control of sexual differentiation and behavior by the doublesex gene in Drosophila melanogaster. Nat Neurosci *13*, 458-466.

S4. Alekseyenko, O.V., Lee, C., and Kravitz, E.A. (2010). Targeted manipulation of serotonergic neurotransmission affects the escalation of aggression in adult male Drosophila melanogaster. PLoS One *5*, e10806.

S5. Ng, M., Roorda, R.D., Lima, S.Q., Zemelman, B.V., Morcillo, P., and Miesenbock, G. (2002). Transmission of olfactory information between three populations of neurons in the antennal lobe of the fly. Neuron *36*, 463-474.

S6. Billeter, J.C., Villella, A., Allendorfer, J.B., Dornan, A.J., Richardson, M., Gailey, D.A., and Goodwin, S.F. (2006). Isoform-specific control of male neuronal differentiation and behavior in Drosophila by the fruitless gene. Curr Biol *16*, 1063-1076.
